# Genetic polymorphisms in circadian negative feedback regulation genes predict overall survival and response to chemotherapy in gastric cancer patients

**DOI:** 10.1038/srep22424

**Published:** 2016-03-01

**Authors:** Falin Qu, Qing Qiao, Nan Wang, Gang Ji, Huadong Zhao, Li He, Haichao Wang, Guoqiang Bao

**Affiliations:** 1 Department of General Surgery, Tangdu Hospital, The Fourth Military Medical University, Xi’an 710038, China; 2 Xijing Hospital of Digestive Disease, The Fourth Military Medical University, Xi’an, 710032 China; 3 Department of Ophthalmology, School of Medicine, Emory University, Atlanta, GA 30322, USA; 4 Department of Emergency Medicine, North Shore University Hospital, Manhasset, NY 11030, USA; 5 93926 Army Hospital of PLA, Hetian 848000, China

## Abstract

Circadian negative feedback loop (CNFL) genes play important roles in cancer development and progression. To evaluate the effects of single nucleotide polymorphisms (SNPs) in CNFL genes on the survival of GC patients, 13 functional SNPs from 5 CNFL genes were genotyped in a cohort of 1030 resected GC patients (704 in the training set, 326 in the validation set) to explore the association of SNPs with overall survival (OS). Among the 13 SNPs, three SNPs (rs1056560 in *CRY1*, rs3027178 in *PER1* and rs228729 in *PER3*) were significantly associated with OS of GC in the training set, and verified in the validation set and pooled analysis. Furthermore, a dose-dependent cumulative effect of these SNPs on GC survival was observed, and survival tree analysis showed higher order interactions between these SNPs. In addition, protective effect conferred by adjuvant chemotherapy (ACT) on GC was observed in patients with variant alleles (TG/GG) of rs1056560, but not in those with homozygous wild (TT) genotype. Functional assay suggested rs1056560 genotypes significantly affect CRY1 expression in cancer cells. Our study presents that SNPs in the CNFL genes may be associated with GC prognosis, and provides the guidance in selecting potential GC patients most likely responsive to ACT.

Gastric cancer (GC) is the second leading cause of cancer-related deaths worldwide, affecting nearly one million people per year^1^. The prognosis of GC patients continues to be dismal, despite the improving surgical and adjuvant treatment approaches, with a 5-year overall survival (OS) less than 25%^2^. Although Tumor-Node-Metastasis (TNM) staging system is the best available clinical prediction of GC prognosis, the long-term outcome remains unoptimistic for GC^3^. Therefore, it is urgent to discover novel biomarkers for more effective prognosis prediction and to subsequently improve therapeutic benefit for GC patients.

Circadian rhythms occur naturally with a periodicity of approximately 24 hours, and play an essential role in regulating the daily rhythms of human physiology and behaviors. The regulation of circadian oscillators occurs through a series of positive/negative transcriptional-translational feedback loops including at least nine core circadian clock genes^4^. Among them, period homolog (PER1, PER2 and PER3) and cryptochrome (CRY1 and CRY2) clock proteins form complexes to negatively inhibit the nuclear transcription activities of CLOCK-BMAL1 and NPAS2-BMAL1^5^, whereas the disruption of circadian rhythms may facilitate tumorigenesis^6^. Therefore, there is a possible link between circadian negative feedback loop (CNFL) genes and several aspects of carcinogenesis including cell proliferation, angiogenesis, apoptosis, metabolism and DNA damage response^7^. Indeed, the altered expression of CNFL genes (*PER1*, *PER2*, *PER3*, *CRY1* and *CRY2*) is frequently observed in various types of human cancers^8^,^9^,^10^, and their abnormal expression may be associated with poor prognosis in several types of cancers, including GC^11^. It was previously unknown whether the variances of CNFL genes could serve as novel prognostic markers to predict overall outcome of GC patients.

Single nucleotide polymorphisms (SNPs) are attractive biomarkers for translational studies due to its easy-detection from blood samples^12^. A number of SNPs associated with cancer risk and prognosis have been identified in human CNFL genes, as documented by large population-based studies^13^. For instance, a synonymous SNP in *PER3* gene, rs2640908, has been found to be correlated with prognosis of hepatocellular carcinoma(HCC) patients with TACE treatment^14^. Similarly, six tagging SNPs in CNFL genes are associated with patients’ susceptibility to prostate cancer^15^. One recent study has reported that four functional SNPs in CNFL genes are associated with shorter OS and relapse-free survival(RFS) in HCC patients after radical surgery^16^. However, the association between the functional SNPs in CNFL genes and the clinical outcomes of GC patients remains not to be determined.

Herein, to test the hypothesis that the polymorphisms of CNFL genes may affect the prognosis and clinical outcome of GC, we assessed the effects of thirteen functional SNPs in *PER1, PER2, PER3, CRY1* and *CRY2* on survival time of 1030 Chinese GC patients (704 in the training set, 326 in the independent validation set) who received radical resection treatment. Additionally, the effect of an identified relevant SNP–*CRY1* rs1056560–on the regulation of gene expression was further tested by an *in vitro* functional assay. To the best of our knowledge, this is the first investigation of the association between SNPs in CNFL genes and the clinical outcome of GC.

## Results

### Distribution of patients’ characteristics and prognosis analysis

This study included 1030 patients with resected gastric adenocarcinoma, and the demographic and clinical characteristics of GC patients were summarized in Supplementary Table 1. The median follow-up time was shorter in the training set (46 months ranging from 6 to 80 months) than in the independent validation set (72 months ranging from 6 to 89 months) due to the late ending date of patient enrollment at the training set. Thus the patients in the training set had lower rates of relapse (58.4%) and death (41.4%) than those in the independent validation set (66.8% and 55.8%, respectively) (*P* = 0.005 and *P* = 0.001). At the latest follow-up, 641 patients (423 and 218 in the training and validation set, respectively) developed relapse and 482 died (300 and 182 in the training and validation set, respectively). There were no differences between the training set and the validation set in terms of age, tumor site, tumor size, TNM stage, differentiation and chemotherapy (*P* value ranging from 0.082 to 0.898). Furthermore, we performed a multivariate analysis of OS and RFS in GC for all the prognostic variables by Cox proportional hazard model. As expected, our data showed that the risk of death for GC was significantly increased as the stage increased in a dose-response manner among training set, validation set and pooled analysis (all *P* for trend <0.001), and a similar result was obtained for risk of recurrence (all *P* for trend <0.001). All patient sets exhibited significant worse OS and RFS in patients with larger tumor size or poor differentiated tumor. In addition, platinum-based adjuvant chemotherapy (ACT) after surgery had significant protective effects on both OS and RFS of GC patients (Supplementary Table 2).

### Association of single SNP with clinical outcome of GC patients

**Figure 1:**
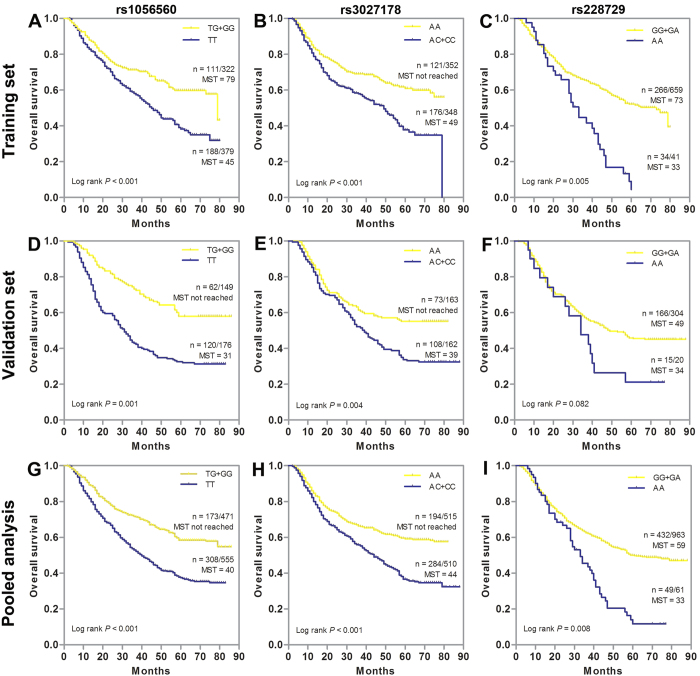
Kaplan-Meier estimates of overall survival (OS) for gastric cancer (GC) patients stratified by genetic variants of circadian negative feedback loop genes. OS of GC patients stratified by rs1056560, rs3027178 and rs228729 in (**A–C**) training set, (**D–F**) validation set and (**G–I**) pooled analysis. MST indicates median event-free survival times (in months). Patient numbers may not add up to 100% of available subjects because of missing genotyping data.

We assessed the association between each individual SNP and clinical outcome using the multivariate Cox proportional hazard model with adjustment for age, sex, tumor site, tumor size, differentiation, TNM stage and chemotherapy under dominant, recessive, and additive models, then presented the results with best-fitting model (Table 1 and Supplementary Table 3). The data analysis showed that three SNPs had significant associations with the OS of GC patients in the training set. Among them, SNP rs1056560 in the *CRY1* gene exhibited a significant protective effect on the OS in GC patients, with a HR of 0.72 (95% CI 0.58–0.88, *P* = 0.021) under the additive model. SNP rs3027178 in the *PER1* gene was significantly associated with an increased death risk in GC patients, with a HR of 1.72 (95% CI 1.19–2.35, *P* = 0.001) under the dominant model. SNP rs228729 in the *PER3* gene, showed a detrimental effects on GC death risk, with HRs of 1.93 (95% CI 1.31–2.85, *P* = 0.003) under the recessive model. These significant results were validated by the internal validation method of bootstrapping. Kaplan-Meier curves analysis also revealed a strong association with OS. Patients with TG/GG genotypes of rs1056560 had better OS than those with TT genotype (*P* < 0.001, Fig. 1A), while patients carrying AC/CC genotypes of rs3027178 had poorer OS than those with AA genotype (*P* < 0.001, Fig. 1B). Besides, patients carrying AA genotype of rs228729 had worse OS than those carrying GA/GG genotypes (*P* = 0.005, Fig. 1C).

**Figure 2:**
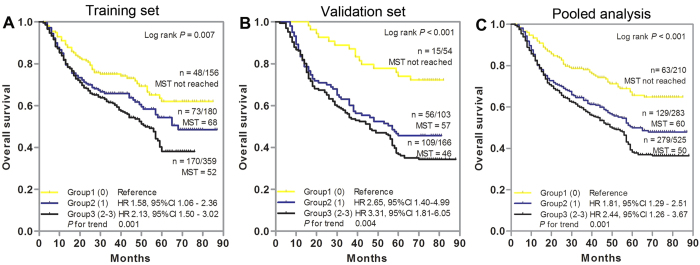
Cumulative analysis of unfavorable genotypes on the overall survival of gastric cancer patients. (**A**) Training set, (**B**) Validation set, (**C**) Pooled analysis. Kaplan-Meier curve and multivariate Cox proportional hazards regression were used to estimate the cumulative effects of unfavorable genotypes. HRs were adjusted by age, sex, tumor site, tumor size, differentiation, TNM stage and adjuvant chemotherapy. Significant SNPs (*P* < 0.05) were included to categorize unfavorable genotype groups. MST indicates median event-free survival times (in months). Patient numbers may not add up to 100% of available subjects because of missing genotyping data.

When another set of 326 GC patients was added, the previously significant associations were replicated in the independent external validation set except for *PER3* rs228729. Multivariate Cox proportional hazards regression analyses demonstrated that *CRY1* rs1056560 and *PER1* rs3027178 both remained significant associations with OS of GC patients, with HRs of 0.74 (95% CI 0.46–0.90; *P* = 0.023) and 1.54 (95% CI 1.07–1.98; *P* = 0.034), respectively. In validation set, although not reaching statistical significance, SNP rs228729 showed a similar trend of association with OS in training set. The pooled analysis of the two patient sets further confirmed the previously observed associations with OS for *CRY1* rs1056560 (HR = 0.65, 95% CI = 0.34–0.87, *P* = 0.001) and for *PER1* rs3027178 (HR = 1.71; 95% CI 1.25–2.34; *P* < 0.001), as well as for *PER3* rs228729 (HR = 1.79, 95% CI = 1.29–2.93, *P* = 0.003). Kaplan-Meier plots suggested a significantly better OS in patients carrying TG/GG genotypes of rs1056560 in validation set (*P* = 0.001, Fig. 2D) and pooled analysis (*P* < 0.001, Fig. 2G). Moreover, patients carrying AC/CC genotypes of rs3027178 showed significantly poor OS than those carrying AA genotype in validation set (*P* = 0.004, Fig. 2E) and pooled analysis (*P* < 0.001, Fig. 2H), while patients carrying AA genotype of rs228729 trended to showed worse OS than those carrying GA/GG genotypes in the validation set (*P* = 0.082, Fig. 2F) and pooled analysis (*P* = 0.008, Fig. 2I). In further stratified analyses, no evident modifying effect on the prognostic significance of SNPs was found in terms of age, sex, tumor site, tumor size, TNM stage and differentiation (data not shown).

### Cumulative effects of unfavorable genotypes on the OS of GC patients

To further evaluate the cumulative effect of multiple SNPs on GC death risk, we combined the unfavorable genotype of each individual SNP and analyzed their associations with OS. The unfavorable genotypes were defined as the wild-type genotype (TT) for rs1056560 in the *CRY1* gene, variant allele-containing genotypes (AC/CC) for rs3027178 in the *PER1* gene, and homozygous variant genotype (AA) for rs228729 in the *PER3* gene. There was a significant dose-response trend for the increased risk of death and reduced OS time with increasing number of unfavorable genotypes in training set (*P* for trend = 0.001, Fig. 2A), validation set (*P* for trend = 0.004, Fig. 2B), and pooled analysis (*P* for trend = 0.001, Fig. 2C). In training set, compared with patients in group 1 (with 0 unfavorable genotype), GC patients had a 1.58-fold increased risk of death (95% CI, 1.06–2.36) in group 2 (with 1 unfavorable genotype), and the risk further increased to 2.13-fold (95% CI, 1.50–3.02) for patients in group 3 (with 2 or 3 unfavorable genotypes) (Fig. 2A). Similar cumulative effect was observed in the independent validation set: compared with the group 1, the group 2 had a 2.65-fold (95% CI 1.40–4.99) increased risk, whereas the group 3 was at a 3.31-fold (95% CI 1.81–6.05) increased risk (Fig. 2B). These significant gene-dosage effects were also confirmed in pooled analysis: in comparison with group 1, patients in group 2 and group 3 exhibited a progressively increased risk of death, with HRs of 1.81 (95% CI 1.29–2.51) and 2.44 (95% CI 1.26–3.67), respectively (Fig. 2C). Kaplan-Meier curve analysis showed a significantly different OS among GC patient groups stratified by number of unfavorable genotypes in training set (*P* = 0.007, Fig. 2A), validation set (*P* < 0.001, Fig. 2B), or pooled analysis (*P* < 0.001, Fig. 2C).

### Higher order gene-gene interactions on the prognosis of GC

**Figure 3:**
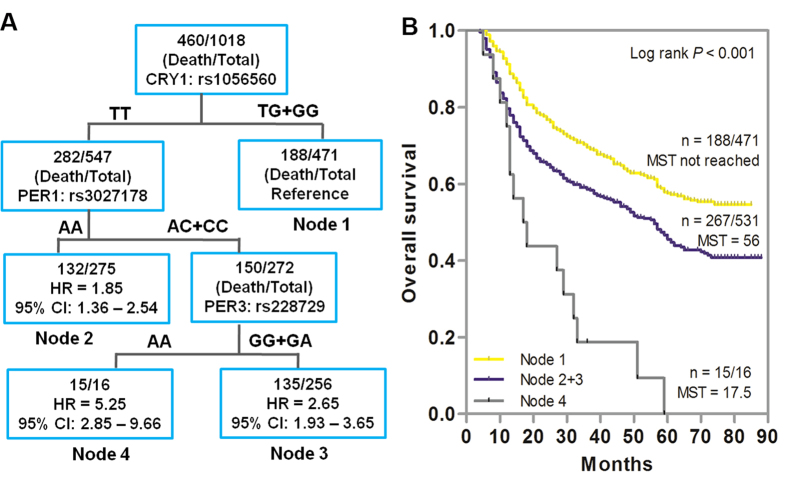
Potential higher-order gene-gene interactions among circadian negative feedback loop gene polymorphisms. (**A**) Tree structure identifying subgroups of patients with different genetic backgrounds. (**B**) Kaplan-Meier survival curves for patients based on survival tree analysis. MST indicates median event-free survival times (in months). Patient numbers may not add up to 100% of available subjects because of missing genotyping data.

Since multiple SNPs in different genetic loci were associated with OS in GC patients, we explored the higher order gene-gene interactions to reveal whether complex interactions among these SNPs could potentially predict the patient outcome by survival tree analysis. As shown in Fig. 3A, three SNPs (*CRY1*: rs1056560, *PER1*: rs3027178, *PER3*: rs228729) exhibited gene-gene interactions, resulting in four terminal nodes with different OS. The initial split on the survival tree was due to rs1056560 in *CRY1* (Node 1), indicating that this SNP was the primary factor contributing to the survival difference in GC patients. The longest survival time was observed in patients of reference group (Node 1), who carried the variant allele-containing genotypes (TG/GG) of *CRY1* rs1056560. The shortest survival time was observed in Node 4 patients, which was composed of individuals with homozygous wild genotype (TT) of *CRY1* rs1056560, variant allele-containing genotypes (AC/CC) of *PER1* rs3027178, and homozygous variant genotype (AA) of *PER3*: rs228729. Kaplan-Meier curve analysis significantly distinguished the patients grouped by survival tree analysis (*P* < 0.001, Fig. 3B).

### The effects of ACT on GC survival modulated by unfavorable SNPs genotypes

Platinum-based ACT is commonly used for stage II and III GC patients to improve survival. However, which patients will benefit from it and the magnitude of benefit across specific subgroups remain largely uncertain. We thus assessed the predictive value of the three confirmed SNPs for OS benefit from platinum-based ACT in stage II and III GC patients. There were 733 stage II and III patients (502 and 231 in the training set and validation set, respectively), among whom 577 received platinum-based ACT (410 and 167 in the training set and validation set, respectively). We first stratified the significant associations of CNFL SNPs and OS of GC by ACT to evaluate whether the significant CNFL SNPs are prognostic markers for all GC or for specific groups of GC patients receiving ACT. As shown in Supplementary Table 4, the reduced risk of death associated with the variant-containing genotypes (TG/GG) of *CRY1* rs1056560 remained significant in those patients receiving platinum-based ACT, with HRs of 0.55 (95% CI = 0.40–0.77, *P* = 0.001) in training set, 0.60 (95% CI = 0.34–0.84, *P* = 0.005) in validation set and 0.58 (95% CI = 0.39–0.83, *P* = 0.005) in pooled analysis, but not in those without ACT. Consistent with the main effect analysis, the associations of *PER1* rs3027178 and *PER3* rs228729 with OS were both significant in GC patients with or without ACT in training set, validation set, and pooled analysis (both *P* < 0.05). However, these significant associations conferred by rs3027178 and rs228729 were more evident in patients with ACT than those without ACT. Taken together, ACT-stratified analysis suggested that the death risks of GC patients associated with potential risk SNPs identified in the present study, especially rs1056560, were more prominent in patients with ACT, indicating a potential modulating effect between these SNPs and ACT on GC outcomes. Therefore, we further evaluated the association between the platinum-based ACT with GC OS, and determined whether such an effect was modulated by the potential risk SNPs identified in the present study. As shown in Table 2, patients subjecting to ACT exhibited a significant lower death risk compared to those without ACT in the training set (HR = 0.70, 95% CI 0.52–0.96, *P* = 0.03), validation set (HR = 0.77, 95% CI 0.61–0.94, *P* = 0.027) and in pooled analysis (HR = 0.71, 95% CI 0.53–0.87, *P* = 0.001). This favorable prognosis conferred by ACT was observed either in patients with the rs3027178 AA genotype or in those with the rs3027178 AC/CC genotypes in both training and validation sets (all *P* < 0.05). Similar results were found in the SNP rs3027178. In comparison, significant protective effect conferred by ACT on GC OS was only observed in patients with variant-containing (TG/GG) genotypes of rs1056560 in the training set (HR = 0.67, 95% CI 0.49–0.95, *P* = 0.023), validation set (HR = 0.65, 95% CI 0.42–0.88, *P* = 0.016) and in pooled analysis (HR = 0.59, 95% CI 0.38–0.80, *P* = 0.007), but not in those with homozygous wild (TT) genotype. These results demonstrated that only patients with variant-containing (TG/GG) genotypes of *CRY1* rs1056560 may benefit from platinum-based ACT, suggesting that *CRY1* rs1056560 may be a promising predictive marker for better response to platinum-based ACT of GC.

### Functional effects of CRY1 rs1056560 on gene expression

**Figure 4:**
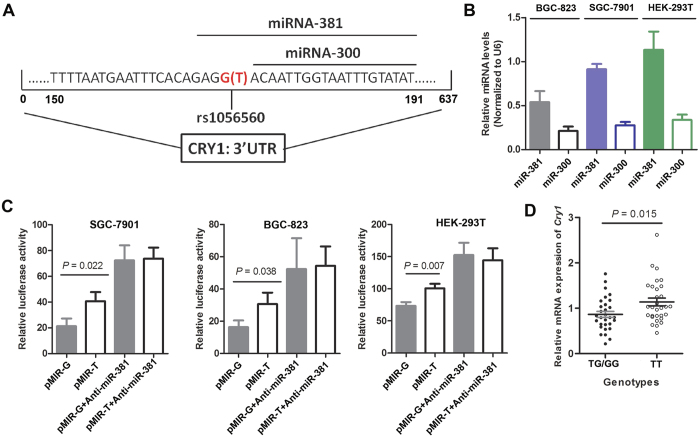
Functional effect of SNP rs1056560 genotypes on gene expression by luciferase reporter assay. (**A**) The sequence including SNP rs1056560 in 3′UTR of *CRY1* gene. (**B**) Relative expression levels of miR-381 and miR-300 in SGC-7901, BGC-823 and HEK-293T cells. (**C**) The effect of SNP rs1056560 genotypes on the expression of *CRY1* gene in SGC-7901, BGC-823 and HEK-293T cells. (**D**) Relative mRNA expression level of *CRY1* gene in 60 GC tissues with different SNP rs1056560 genotypes. Recombinant vector (pMIR-rs1056560-T or pMIR-rs1056560-G) and pTL-TK were co-transfected into SGC-7901, BGC-823 and HEK-293T cells. Data are expressed as mean ± standard deviation (SD) of three independent experiments. Student’s *t* test was used to examine statistical difference.

Bioinformatics analysis (http://www.microrna.org/microrna/home.do) showed a close proximity of *CRY1* rs1056560 to two predicted microRNA binding sites (hsa-miR-381 and has-miR-300)^17^ (Fig. 4A). We further confirmed the expression of miR-381 and miR-300 in SGC-7901, BGC-823 and HEK-293T cell lines, and found that miR-381 had a relatively higher expression level (Fig. 4B). To determine whether the genotypes of SNP rs1056560 in the 3′UTR of the *CRY1* gene could alter gene expression, three cell lines were transfected with luciferase reporter plasmids containing either the wild (TT) or variant (GG) genotype of SNP rs1056560 (Fig. 4C). Our results demonstrated that SNP rs1056560 had a significant influence on the normalized luciferase activity in all transfected cells. Compared to cells transfected with constructs carrying wild genotype (TT) of SNP rs1056560, cells transfected with constructs carrying variant genotype (GG) exhibited a significant decreased luciferase activity. We also evaluated the effect of anti-miR-381 on the luciferase activity of reporter plasmids. The results showed that anti-miR-381 significantly increased the luciferase activity of two UTR constructs (p-MIR-T and p-MIR-G) in all cell lines and eliminated the differences of luciferase activity between two reporter plasmids. Moreover, we used quantitative real-time RT-PCR to investigate the effect of SNP rs1056560 genotypes on the mRNA expression of *CRY1* in 60 GC tissues (30 with TT genotype and 30 with TG/GG genotypes) from validation set. As shown in Fig. 4D, we found that mRNA expression level of *CRY1* was significantly higher in carriers with wild (TT) genotype of rs1056560 than those who carried variant (TG/GG) genotypes (1.14 ± 0.47 *vs.* 0.86 ± 0.47, *P* = 0.015).

## Discussion

In the present study, we investigated the association of thirteen functional SNPs in several CNFL genes with the prognosis of GC patients by a two-stage analysis of training and validation sets. We demonstrated that three SNPs, rs1056560 in *CRY1*, rs3027178 in *PER1* and rs228729 in *PER3*, were significantly associated with the OS in the training set of 704 GC patients. When an independent validation set of 326 GC patients was added, the significant associations were replicated, reaching an even more robust statistical significance in the pooled analysis. Furthermore, we identified a significant cumulative death risk associated with an increasing number of unfavorable genotypes. Further survival tree analysis revealed SNP rs1056560 in *CRY1* gene as the primary factor contributing to improved GC patients’ survival. More importantly, carriers with TG/GG (but not the TT) genotypes of rs1056560 gained significant survival benefit from platinum-based ACT, and functional assay indicated that the genotypes of SNP rs1056560 had a significant influence on *CRY1* mRNA expression both *in vivo* and *in vitro*. To the best of our knowledge, this is the first report on the association between SNPs in CNFL genes and GC prognosis.

Our study now provide compelling evidence to support the hypothesis that the disruption of circadian rhythms in humans may be associated with cancer development and tumor progression. It also supports previous epidemiological studies that the disruption of circadian rhythms in shift workers is accompanied by an increase in cancer risk^18^,^19^,^20^,^21^ and poor cancer prognosis^22^, and agrees with multiple animal and clinical studies that cancer development and progression is closely associated with the loss of circadian homeostasis caused by aberrant CNFL genes. In mice deficient or defective in PER proteins (e.g., PER1, PER2, or PER3), apoptotic cell death is attenuated, and tumor incidence and susceptibility to radiation-induced cancer is increased^23^,^24^,^25^,^26^,^27^,^28^. In contrary, the overexpression of these PER (e.g., PER1) proteins lead to inhibition of cancer cells growth *in vitro* and *in vivo*, and sensitization of cancer cells to DNA damage-induced apoptosis. In contrast to *PER* genes, the loss of *CRY* genes significantly reduces cancer risk^29^. Consistent with these results, the altered expression of CNFL genes are frequently observed in human cancers. For example, Cheng-Ming Hsu *et al.*^30^ found that the expression of CNFL genes were significantly decreased in head and neck squamous cell carcinoma (HNSCC), and lower expression of *PER1* and *PER3* might relate to aggressive phenotypes and poor survival of HNSCC. In contrast, the up-regulated expression of *CRY1* in colorectal cancer is positively correlated with poor patient outcomes^31^. However, *CRY2* is significantly diminished among those suffering from malignancies compared with healthy individuals^8^,^32^. Taken together, these lines of evidences highlight the essential role of CNFL genes in the etiology and clinical outcome of cancers. Therefore, CNFL genes may be useful biomarkers for prognosis prediction and a potential therapeutic target for cancer therapy.

Genetic variants such as SNPs often play an important role in the regulation of gene expression and protein functions^33^. However, the effects of SNPs in CNFL genes on the possible cancer risk and prognosis remains poorly elucidated. It has been suggested that SNPs in CNFL genes (including rs3027178 in *PER1*, rs228669 and rs2640908 in *PER3*, and rs3809236 in *CRY1*) confer susceptibility to various cancers, just as prostate cancer^15^, breast cancer^34^, non-Hodgkin’s lymphoma^35^, and liver cancer^14^,^16^. Here we identified the survival predicting value of three SNPs (including rs3027178 in *PER1*, rs228729 in *PER3*, and rs1056560 in *CRY1*) of CNFL genes for patients with GC.

Of particular interest, the SNP rs1056560 within *CRY1* gene was found to be associated with an increased OS in GC patients, and a significantly better OS was observed in the patients with the variant-containing (TG/GG) genotypes than those with the homozygous wild-type (TT) genotype. This finding is intriguing because the SNP rs1056560 is located in the 3′UTR region of *CRY1* gene, and have the potential to influence its gene expression. Indeed, our bioinformatics analysis (http://www.microrna.org/microrna/home.do) revealed that rs1056560 is within the has-miR-381 binding site and simultaneously close to the has-miR-300 binding site^17^, suggesting that such a variation at this position may have an effect on mRNA stability and binding activity to microRNAs, thereby impacting mRNA cleavage or translational efficiency^36^. Furthermore, our functional assay supported the potential impact of rs1056560 on the post-transcriptional regulation of *CRY1* gene by miR-381. We further evaluated the *CRY1* mRNA expression in 60 GC tissues with genotype data of SNP rs1056560 and found that the tissues carrying variant-containing (TG/GG) genotypes had significantly decreased *CRY1* mRNA expression levels compared to those with homozygous wild (TT) genotype, indicating that the favorable prognostic effect conferred by TG/GG genotypes of rs1056560 was related to down-regulating *CRY1* expression. Our findings were consistent with a previous report by Yu *et al.*^31^ that higher expression of *CRY1* correlated positively with poor outcomes in CRC patient, as well as in GC^11^. Taken together, the evidence in both the current and previous studies suggested a direct causal role of rs1056560 on GC prognosis.

The association of *CRY1* rs1056560 with increased OS is of particular interest, especially in relation to the widely employed platinum-based ACT. Platinum-based ACT is commonly used as an adjuvant treatment for GC patients after surgery. However, the survival benefit from this therapy remains heterogeneous, which are likely influenced by the genetic background and tumor status of the patients. It has been suggested that circadian rhythms may modulate the effects of cancer treatments such as ACT^37^. Therefore, we further conducted stratified analyses to determine whether the significant associations observed in the main effects analysis were modulated by ACT. We found that the death risk of GC associated with *CRY1* rs1056560 was more evident in patients receiving ACT than those without ACT, which were evidenced by the lower HRs and more significant *P* values in ACT-treated patients. In addition, the patients without ACT exhibited an increased HR and *P* value for all SNPs, suggesting potential interactions between the genotypes of *CRY1* rs1056560 and ACT in the modulation of GC survival. Hence, we further demonstrated a protective effect conferred by ACT on patients OS, which was significantly stratified by rs1056560. Our data suggested that the favorable prognosis conferred by ACT was only observed in patients carrying variant-containing (TG/GG) genotypes of rs1056560, but not in those with homozygous wild (TT) genotype. Collectively, these data suggested that *CRY1* rs1056560 might modulate the response of GC patients to the platinum-based ACT. Our finding is in agreement with a previous animal study showing that the decreasing *CRY1* expression renders animals more sensitive to platinum-based ACT cancer therapy^38^. We also found interesting associations between the *PER1* rs3027178 polymorphism and a shorter OS in all population sets, as well as the *PER3* rs228729 polymorphisms and death risk only in the training set and pooled analysis. Future larger scale clinical studies are needed to confirm the roles of these two SNPs on GC survival. Their functional effects on cancer prognosis could also be of interest since both *PER1* and *PER3* belong to core circadian genes, which are involved in cancer development and progression. However, up to date, no studies have been conducted to assessed the molecular functions of rs3207178 and rs228729. Silicon analysis showed that SNPs rs228729 is located in the transcription factor binding region of *PER3*, which may regulate the expression of *PER3* gene. As predicted on http://snpinfo.niehs.nih.gov/snpfunc.htm, SNPs rs3027178 is located in the exonic splicing enhancer (ESE) region *of PER1*, which may affect the sequence of mRNA, leading to variations in protein structures and functions^39^. Based on above silicon analysis, we hypothesized that rs3207178 and rs228729 might affect the prognosis of GC patients through regulating cell proliferation and apoptotic cell death by modulating the expression and activity of *PER1* and *PER3*. Further molecular characterizations are needed to validate the functions of these two SNPs in GC prognosis. Moreover, to the best of our knowledge, neither of rs3207178 and rs228729 were evaluated for their possible modulation in relationship with platinum-based ACT. Our data from stratified analysis suggested that both rs3207178 and rs228729 might not have obvious modifying effects on the favorable prognosis of platinum-based ACT. Further confirmation is warranted using larger samples in future studies.

Single SNP often provides a modest or undetectable effect, whereas the amplified effects of combined multiple SNPs in the same pathway may enhance predictive power. Therefore, we performed cumulative effect analysis by counting the number of unfavorable genotypes to investigate the combined effects of these CNFL SNPs on the OS of GC. We identified a significant trend of poor OS with an increasing number of unfavorable genotypes in a dose-dependent manner. There is important clinical significance to improve the prognosis assessment and treatment decision of GC patients by incorporating these SNPs into the current tumor staging systems. In addition, GC is a multistep and multifactorial disease, we thus conducted survival tree analysis to observe the higher order gene-gene interactions among SNPs in CNFL genes and their effects on OS in GC patients. We found that SNP rs1056560 in *CRY1* gene as the primary split in the survival tree analysis exhibited the strongest influence on GC patients’ OS. These results suggest that the clinical progression or remission of GC is a polygenic process and *CRY1* rs1056560 may be a driver SNP during GC evolution.

Our study has several strengths. First, the patient population is enrolled from Shaanxi and adjacent areas. This region is highly attractive in conducting population-based research due to the geographical stability with low mobility rate. Second, the relatively large population size enrolled in present study allowed us to conduct stage-stratified analysis in the training and validation sets, which limited the confounding factors of tumor and treatment heterogeneity. One major limitation of this study is that the relative small population in the validation set may result in false-negatives, which may partially explain why rs228729 was not validated in the independent validation set. Moreover, since our study was restricted to Han Chinese, future studies in larger populations and other ethnics are warranted.

In conclusion, our study for the first time demonstrates that *CRY1* rs1056560, *PER1* rs3027178 and *PER3* rs228729 are associated with the OS of GC patients and can be used to predict the prognosis of GC individually and collectively. Furthermore, *CRY1* 1056560 may be used as predicting marker to facilitate physicians in making individualized treatment decisions. Independent studies are needed to validate these findings before their clinical applications.

## Methods

### Study population

Between January 2008 and June 2013, a total of 1094 Han Chinese patients with primary GC were recruited from Tangdu Hospital and Xijing Hospital of Digestive Disease, affiliated to the Fourth Military Medical University, in Xi’an, China. All GC cases had no previous history of other cancers or any preoperative anticancer treatment or blood transfusion within 3 months before operation. There were no age, sex, or disease stage restrictions for case recruitment. All patients were newly diagnosed and histologically confirmed to be adenocarcinoma. In the present study, we excluded a total of 64 patients, including 16 patients who did not undergo surgery or only received palliative operation, 37 who had incomplete clinical information or failed follow-up, 5 who died within 1 months after surgery, and 6 who had poor quality of DNA sample. Finally, 1030 patients with radically resected gastric adenocarcinoma were included in the present study and successfully genotyped. Among them, 704 patients from Department of general surgery, Tangdu Hospital between October 2008 and June 2013 were used as the training set for this study. 326 patients from Xijing Hospital of Digestive Disease between January 2008 and December 2010 were used as an independent validation set. This study was approved by the Ethic Committee of The Fourth Military Medical University and a signed informed consent was obtained from each participant. The methods were carried out in accordance with the approved guidelines.

### Demographic and clinical data

Demographic and clinical data were collected through in-person interviews at the time of initial visit or follow-up in the clinics, medical records, or consultation with treating physicians by well-trained clinical research specialists, including age, sex, ethnicity, residential region, time of diagnosis, time of surgery and/or chemotherapies, time of relapse and/or death, tumor stage, differentiation, histological type, tumor site, lymph node invasiveness, and treatment protocol. Follow-up information was updated at 6-month intervals through on-site interview, telephone calling, or medical records. The latest follow-up date was June 2015 and the median follow-up duration was 51 months (range 6–89 months). The percentage of patient lost during follow-up was 9.8%. OS was defined as the time from surgery to GC-specific death. RFS was defined as the time from surgery to the date of the first recurrence or distant metastasis of GC. Patients alive at the last follow-up were censored.

For each GC patients, 5 mL venous blood was collected before surgery and used for DNA extraction by using the E.Z.N.A. blood DNA Midi Kit (Omega Bio-Tek, Norcross, GA, USA) in the laboratory. Sixty gastric cancerous tissues were simultaneously gathered from the validation set for real-time quantitative reverse transcription PCR assay.

### SNP selection and genotyping

Functional SNPs in CNFL genes (*CRY1, CRY2, PER1, PER2* and *PER3*) were selected using a set of web-based SNP selection tools (http://snpinfo.niehs.nih.gov/snpinfo/snpfunc.htm)^40^. Potential functional SNPs were included to meet the following criteria: (1) Validated SNPs with minor allele frequency >5% in the Asian population; (2) SNPs in miRNA binding sites of 3′ untranslated region (UTR); (3) SNPs in the transcription factor binding site of the 5′ flanking region (3000 bp upstream from the transcription start site); (4) SNPs in splice sites and non-synonymous SNPs in exons; (5) In the case of multiple functional SNPs within the same haplotype block and the linkage coefficient *r*^2^ > 0.8, only 1 SNP was included. Finally, a total of 13 functional SNPs in CNFL genes were selected, including 1 non-synonymous SNPs (rs934945 in *PER2*), 3 SNPs in potential transcription factor binding sites (rs172933 and rs228729 in *PER3*, and rs3809236 in *CRY1*), 6 SNPs in potential splice sites (rs3027178 and rs2735611 in *PER1*, rs2304669 in *PER2*, rs228669, rs2640908 and rs2859390 in *PER3*) and 3 SNPs in potential miRNA-binding sites (rs1056560 in *CRY1*, rs2292910 and rs6798 in *CRY2*). Genotyping was carried out using Sequenom iPLEX genotyping system (Sequenom Inc., San Diego, CA, USA) according to the manufacturer’s protocol. Strict quality controls were performed in each assay during genotyping, and SNP with a call rate >98% was included for further analysis.

### Functional assay

The dual-luciferase reporter assay was used to investigate functional effects of rs1056560 located in the 3′UTR of *CRY1* gene. First, hsa-miR-381, hsa-miR-300 and U6 microRNA levels were detected using TaqMan microRNA Reverse Transcription kit (Applied Biosystem, Foster City, CA, USA). Human GC cell lines SGC-7901, BGC-823 and embryonic kidney cell line 293 (HEK293) were purchased from the Type Culture Collection of the Chinese Academy of Sciences (Shanghai, China). Briefly, 200 ng of total RNA from SGC-7901, BGC-823, or HEK-293T was used for primer-specific reverse transcription (RT), and 2 μL of the RT product was used for subsequent quantitative PCR. Then, 51-bp double-strand DNA carrying either wild genotype or variant genotype of rs1056560 were synthesized and cloned into the pMIR-REPORT vector (Ambion, Austin, TX, USA) using restriction enzymes SpeI and HindIII (Takara, Dalian, China). All the constructs were confirmed by DNA sequencing. Three cell lines were co-transfected with either pMIR-rs1056560-T or pMIR-rs1056560-G (200 ng/well) with or without anti-miR-381 (Applied Biosystems, Foster City, CA, USA) and the internal control reporter plasmid pRLTK (Promega, Madison, WI, USA) (20 ng/well) using Lipofectamine 2000 (Invitrogen, Carlsbad, CA, USA). After 48 h, the cells were collected to determine luciferase activity using a dual-luciferase reporter assay system kit (Promega, Madison, WI, USA) with a luminometer (Tecan, Mannedorf, Switzerland). All transfections were performed in triplicates, and all experiments were independently repeated three times.

To further assess the effect of SNP rs1056560 genotypes on *CRY1* mRNA expression, total RNA was isolated from 60 GC tissue samples (30 with TT genotype and 30 with TG/GG genotypes of rs1056560) according to the manufacturer’s instructions. Then, cDNA were synthesized using PrimeScript RT reagent kit (Takara, Dalian, China). The quantitative real-time reverse transcription PCR (RT-PCR) was performed as previously described using following *CRY1* primers: forward, 5′-CTTGATGCAGATTGGAGCAT-3′; reverse, 5′-CCATTGGGATCTGTTCTCCT-3′[11], and hGAPDH was used as an internal control. Relative expression of *CRY1* mRNA levels was determined using the relative quantification method and 2^−∆∆Ct^ analysis.

### Statistical analysis

Statistics analyses were carried out using the IBM SPSS Statistics 19.0 software (IBM). Normally distributed continuous variables were expressed as mean ± SD, while abnormally distributed continuous variables were expressed as median and range. Pearson’s χ^2^-test was used to test the differences of categorical variables. Student’s *t*-test was used to analyze the difference of normally distributed continuous variables between two groups, while Mann-Whitney U test was employed for the comparison of abnormally distributed continuous variables. Multivariate Cox proportional hazard regression model was applied to assess the effect of individual SNP and patients’ characteristics on overall or recurrence-free survival. Hazard ratios (HRs) and 95% confidence intervals (CIs) were estimated with adjustment for age, sex, tumor site, tumor size, differentiation, TNM stage and chemotherapy. The main analyses were performed under three genetic models, including dominant (homozygous variant plus heterozygous genotypes *vs.* homozygous wild type), additive (homozygous variant *vs.* heterozygous *vs.* homozygous wild type, showing as *P* for trend), and recessive (homozygous variant *vs.* heterozygous plus homozygous wild-type genotypes) models. The best-fitting model was defined as that with the smallest *P* value for the association analysis. The results of the main effect analyses were internally validated by the bootstrap re-sampling method^41^. One hundred bootstrap samples that were drawn from the original data set were generated for each analysis, and we counted the number of times as *P* value was <0.05. Kaplan-Meier curves and log-rank tests were also used to evaluate effect of the individual SNPs on survival time.

The cumulative effects of SNPs with significant association (*P* for the best-fitting model <0.05) with the survival of GC patients were evaluated by counting the number of unfavorable genotypes in each subject. Using a multivariable Cox proportional hazard regression model, HRs and 95% CIs for all groups were calculated and the group of patients carrying no unfavorable genotype was defined as the reference group. Survival tree analyses were used to determine the higher-order gene-gene interactions, which was performed by the STREE program (http://c2s2.yale.edu/software/stree/) using recursive-partitioning to identify subgroups of individuals at higher risk. The tree starts with the root node that includes all the study participants and uses a log-rank statistic to select the optimal split that divides patients into better and worse survival. The recursive procedure continues to produce subsequent nodes that are more homogeneous (with respect to survival) than the original node. The final model is a tree structure with many binary splits, and each terminal node represents a group of patients with different survival patterns based on distinct genotype combinations. HRs and 95% CIs were calculated for terminal nodes using multivariate Cox proportional hazard models after adjusting for host variables. Statistics significance was set at a level of 0.05 and all *P* values reported in this study were two sided.

## Additional Information

**How to cite this article**: Qu, F. *et al.* Genetic polymorphisms in circadian negative feedback regulation genes predict overall survival and response to chemotherapy in gastric cancer patients. *Sci. Rep.* **6**, 22424; doi: 10.1038/srep22424 (2016).

## Supplementary Material

Supplementary Tables

## Figures and Tables

**Table 1:** Association of polymorphisms in circadian negative-feedback loop genes with overall survival in GC patients.

Gene	SNP	Predicted function	Best Fitting Model	Training set			Validation set		Pooled analysis	
				HR^a^ (95% CI)	*P*	Bootstrap^b^ (*P* < 0.05)	HR^a^ (95% CI)	*P*	HR^a^ (95% CI)	*P*
CRY1	rs3809236	TFBS	Dominant	0.85 (0.64–1.09)	0.501		0.96 (0.75–1.21)	0.713	0.92 (0.69–1.28)	0.579
	rs1056560	miRNA	Additive	**0.72 (0.58**–**0.88)**	**0.021**	96	**0.74 (0.46**–**0.90)**	**0.023**	**0.65 (0.34**–**0.87)**	**0.001**
CRY2	rs6798	TFBS	Dominant	0.88 (0.67–1.16)	0.422		0.97 (0.57–1.61)	0.869	0.95 (0.64–1.42)	0.287
	rs2292910	miRNA	Dominant	0.95 (0.76–1.19)	0.652		1.12 (0.51–2.50)	0.725	1.07 (0.72–1.58)	0.695
PER1	rs2735611	Splicing	Dominant	0.87 (0.68–1.11)	0.275		0.92 (0.58–1.48)	0. 841	0.95 (0.65–1.57)	0.881
	rs3027178	Splicing	Dominant	**1.72 (1.19**–**2.35)**	**0.001**	100	**1.54 (1.07**–**1.98)**	**0.034**	**1.71 (1.25**–**2.34)**	**<0.001**
PER2	rs2304669	Splicing	Dominant	0.92 (0.70–1.20)	0.403		1.12 (0.83–1.58)	0.612	1.16 (0.70–1.52)	0.326
	rs934945	nsSNP	Dominant	0.76 (0.56–1.25)	0.482		0.91 (0.62–1.47)	0.862	0.89 (0.58–1.35)	0.933
PER3	rs228729	TFBS	Recessive	**1.93 (1.31**–**2.85)**	**0.003**	98	1.39 (0.82–1.97)	0.170	**1.79 (1.29**–**2.93)**	**0.003**
	rs228669	Splicing	Dominant	1.15 (0.95–1.40)	0.391		1.14 (0.79–1.42)	0.506	1.19 (0.86–1.51)	0.275
	rs2640908	Splicing	Recessive	1.44 (0.98–1.96)	0.076		1.31 (0.93–1.86)	0.127	1.27 (0.95–1.62)	0.113
	rs172933	TFBS	Recessive	1.21 (0.74–1.44)	0.203		1.17 (0.64–1.51)	0.320	1.25 (0.98–1.57)	0.062
	rs2859390	Splicing	Dominant	0.92 (0.72–1.18)	0.516		0.98 (0.73–1.31)	0.962	0.94 (0.69–1.43)	0.898

Note: The significant values were shown in boldface (*P* < 0.05).

HR indicates hazard ratio; CI, confidence interval; TFBS, transcription factor binding site.

^a^Adjusted by age, sex, tumor site, tumor size, differentiation, TNM stage and chemotherapy where appropriate.

^b^Bootstrap analysis was performed using 100 replicates to determine statistics support.

**Table 2:** Modulating effects of adjuvant chemotherapy (ACT) on gastric cancer overall survival by SNPs in circadian negative feedback loop genes.

	Training set			Validation set			Pooled analysis		
Genotype and variables	Deaths/Total^a^	HR (95%CI)^b^	*P*	Deaths/Total^a^	HR (95%CI)^b^	*P*	Deaths/Total^a^	HR (95%CI)^b^	*P*
In all patients									
No ACT	58/92	Reference		51/64	Reference		109/156	Reference	
ACT	171/410	0.70 (0.52–0.96)	0.03	80/167	0.77 (0.61–0.94)	0.027	251/577	0.71 (0.53–0.87)	0.001
In patients with TT genotype of rs1056560									
No ACT	36/50	Reference		32/35	Reference		68/85	Reference	
ACT	116/221	0.83 (0.55–1.26)	0.199	53/88	0.85 (0.48–1.17)	0.163	169/309	0.78 (0.46–1.08)	0.096
In patients with TG/GG genotype of rs1056560									
No ACT	21/41	Reference		19/29	Reference		40/70	Reference	
ACT	55/189	0.67 (0.49–0.95)	0.023	27/79	0.65 (0.42–0.88)	0.016	82/268	0.59 (0.38–0.80)	0.007
In patients with AA genotype of rs3027178									
No ACT	21/47	Reference		22/33	Reference		43/80	Reference	
ACT	69/206	0.78 (0.54–0.99)	0.046	31/86	0.75 (0.45–0.93)	0.034	100/292	0.73 (0.42–0.91)	0.031
In patients with AC/CC genotype of rs3027178									
No ACT	35/43	Reference		29/31	Reference		64/74	Reference	
ACT	101/203	0.74 (0.49–0.97)	0.045	49/81	0.79 (0.42–0.98)	0.046	150/284	0.68 (0.39–0.86)	0.012
In patients with GG/GA genotype of rs228729									
No ACT	53/86	Reference		47/60	Reference		100/146	Reference	
ACT	156/385	0.65 (0.41–0.88)	0.023	72/156	0.68 (0.39–0.89)	0.028	228/541	0.61 (0.34–0.82)	0.001
In patients with AA genotype of rs228729									
No ACT	5/6	Reference		4/4	Reference		9/10	Reference	
ACT	15/25	0.80 (0.62–1.00)	0.05	7/10	0.83 (0.55–1.02)	0.068	22/35	0.78 (0.53–0.99)	0.05

Note: HR, hazard ratio; CI, confidence interval.

^a^Only including stage II and stage III GC patients.Numbers may not add up to 100% of available subjects because of missing genotyping data.

^b^Adjusted by age, sex, tumor site, tumor size, differentiation, TNM stage, and chemotherapy where appropriate.
