# Supplementary material for: Genetic polymorphisms in circadian negative feedback regulation genes predict overall survival and response to chemotherapy in gastric cancer patients
Source: Sci Rep. 2016 Mar 1;6:22424. doi: 10.1038/srep22424 (PMC4772484; doi:10.1038/srep22424)
Supplement: Supplementary Tables [file srep22424-s1.doc]

**Genetic polymorphisms in circadian negative feedback regulation genes predict overall survival and response to chemotherapy in gastric cancer patients**

Falin Qu, Qing Qiao, Nan Wang, Gang Ji, Huadong Zhao, Li He, Haichao Wang, Guoqiang Bao

| **Supplementary Table 1.** Selected demographic and clinical characteristics of gastric cancer patient population. | | | | |
| --- | --- | --- | --- | --- |
| Variables | Training set (n = 704) No. (%) | Validation set (n = 326) No. (%) | *P* value | Total (n = 1030)  No. (%) |
| Sex |  |  |  |  |
| Male | 544 (77.3) | 243 (74.5) |  | 787 (76.4) |
| Female | 160 (22.7) | 83 (25.5) | 0.337^a^ | 243 (33.6) |
| Tumor site |  |  |  |  |
| Proximal | 196 (27.8) | 98 (30.1) |  | 294 (28.5) |
| Body | 257 (36.5) | 104 (31.9) |  | 361 (35.0) |
| Distal | 251 (35.7) | 124 (38.0) | 0.353^a^ | 375 (36.5) |
| Tumor size |  |  |  |  |
| ≤ 5 cm | 541 (76.8) | 234 (71.8) |  | 775 (75.2) |
| > 5 cm | 163 (23.2) | 92 (28.2) | 0.082^a^ | 255 (24.8) |
| TNM stage |  |  |  |  |
| I | 147 (20.9) | 64 (19.6) |  | 211 (20.5) |
| II | 339 (48.2) | 157 (48.2) |  | 496 (48.2) |
| III | 163 (23.2) | 74 (22.7) |  | 237 (23.0) |
| IV | 55 (7.7) | 31 (9.5) | 0.811^a^ | 86 (8.3) |
| Differentiation |  |  |  |  |
| Well | 169 (24.0) | 75 (23.0) |  | 244 (23.7) |
| Moderate | 185 (26.3) | 93 (28.5) |  | 278 (27.0) |
| Poor | 336 (47.7) | 152 (46.6) |  | 488 (47.4) |
| Unknown | 14 (2.0) | 6 (1.9) | 0.898^a^ | 20 (1.9) |
| Chemotherapy |  |  |  |  |
| No | 294 (41.8) | 159 (48.8) |  | 453 (44.0) |
| Yes | 410 (58.2) | 167 (51.2) | 0.098^a^ | 577 (56.0) |
| Relapse |  |  |  |  |
| Yes | 423 (58.4) | 218 (66.8) |  | 641 (62.2) |
| No | 301 (41.6) | 106 (33.2) | 0.006^a^ | 389 (37.8) |
| Death |  |  |  |  |
| Yes | 300 (41.4) | 182 (55.8) |  | 482 (46.8) |
| No | 424 (58.6) | 144 (44.2) | 0.001^a^ | 548 (53.2) |
| Age (years), median (range) | 57 (20 - 83) | 57 (21 - 81) | 0.793^b^ | 57 (20 - 83) |
| Follow-up time (months), median (range) | 46 (6 - 80) | 72 (6 - 89) | <0.001^b^ | 51 (6 - 89) |

Notes: TNM indicates tumor-node-metastasis; Significant P value was in bold.

^a^ The P values were calculated using a Pearson Chi-Square test.

^b^ The P values were calculated using a Mann-Whitney U test.

| **Supplementary Table 2.** Distribution of patients' characteristics and prognosis analysis in the Training set and the Validation set. | | | | | | | | | | | | | | |
| --- | --- | --- | --- | --- | --- | --- | --- | --- | --- | --- | --- | --- | --- | --- |
| Variables | Training set (n = 704) | | | |  | Validation set (n = 326) | | | |  | Pooled analysis (n = 1030) | | | |
|  | Deaths/  Total  300/704 | HR^a^ (95% CI) | Relapse/  Total  423/704 | HR^a^ (95% CI) |  | Deaths/  Total 182/326 | HR^a^ (95% CI) | Relapse/  Total 218/326 | HR^a^ (95% CI) |  | Deaths/  Total 482/1030 | HR^a^ (95% CI) | Relapse/  Total 641/1030 | HR^a^ (95% CI) |
| Age |  |  |  |  |  |  |  |  |  |  |  |  |  |  |
| ≤57 | 146/339 | Reference | 194/339 | Reference |  | 90/160 | Reference | 108/160 | Reference |  | 236/499 | Reference | 302/499 | Reference |
| >57 | 154/365 | 0.99 (0.78 – 1.26) | 229/365 | 1.14 (0.89 – 1.36) |  | 92/166 | 0.86 (0.64 – 1.31) | 110/166 | 0.95 (0.67 – 1.29) |  | 246/531 | 0.91 (0.73 – 1.28) | 339/531 | 1.08 (0.76 – 1.35) |
| Sex |  |  |  |  |  |  |  |  |  |  |  |  |  |  |
| Male | 243/544 | Reference | 336/544 | Reference |  | 136/243 | Reference | 156/243 | Reference |  | 379/787 | Reference | 492/787 | Reference |
| Female | 57/160 | 0.77 (0.57 – 1.12) | 87/160 | 0.82 (0.63 – 1.07) |  | 46/83 | 0.88 (0.62 – 1.24) | 62/83 | 0.98 (0.74 – 1.35 |  | 103/243 | 0.86 (0.60 – 1.26) | 149/243 | 0.91 (0.73 – 1.28) |
| Tumor site |  |  |  |  |  |  |  |  |  |  |  |  |  |  |
| Proximal | 91/196 | Reference | 118/196 | Reference |  | 57/98 | Reference | 67/98 | Reference |  | 148/294 | Reference | 185/294 | Reference |
| Body | 103/257 | 0.86 (0.64 – 1.17) | 149/257 | 0.93 (0.72 – 1.22) |  | 58/104 | 0.98 (0.72 – 1.26) | 68/104 | 0.99 (0.70 – 1.31) |  | 161/361 | 0.89 (0.58 – 1.32) | 217/361 | 0.96 (0.69 – 1.40) |
| Distal | 106/251 | 0.91 (0.67 – 1.22) | 156/251 | 1.09 (0.84 – 1.42) |  | 67/124 | 0.96 (0.65 – 1.38) | 83/124 | 0.97 (0.75 – 1.29) |  | 173/375 | 0.93 (0.70 – 1.27) | 239/375 | 1.01 (0.78 – 1.25) |
| Tumor size |  |  |  |  |  |  |  |  |  |  |  |  |  |  |
| ≤5 cm | 208/541 | Reference | 300/541 | Reference |  | 117/234 | Reference | 132/234 | Reference |  | 325/775 | Reference | 432/775 | Reference |
| >5 cm | 92/163 | **1.64 (1.05 - 2.47)** | 123/163 | **1.52 (1.02 – 1.93)** |  | 65/92 | **1.94 (1.13 - 3.61)** | 86/92 | **2.28 (1.26 – 4.65)** |  | 157/255 | **1.79 (1.08 - 2.84)** | 209/255 | **1.65 (1.05 – 2.26)** |
| Differentiation^b^ |  |  |  |  |  |  |  |  |  |  |  |  |  |  |
| Well/moderate | 122/354 | Reference | 184/354 | Reference |  | 81/168 | Reference | 97/168 | Reference |  | 203/522 | Reference | 281/522 | Reference |
| Poor | 164/336 | **1.62 (1.04 – 2.35)** | 229/336 | **1.64 (1.03 – 2.78)** |  | 97/152 | **1.55 (1.01 – 2.84)** | 116/152 | **1.83 (1.16 – 3.93)** |  | 261/488 | **1.67 (1.08 – 2.66)** | 345/488 | **1.76 (1.10 – 3.07)** |
| TNM stage |  |  |  |  |  |  |  |  |  |  |  |  |  |  |
| I | 35/147 | Reference | 59/147 | Reference |  | 26/64 | Reference | 31/64 | Reference |  | 61/211 | Reference | 90/211 | Reference |
| II | 145/339 | **1.59 (1.09 – 2.31)** | 205/339 | **1.43 (1.04 – 1.95)** |  | 79/157 | **1.45 (1.02 – 1.96)** | 89/157 | 1.41 (0.99 – 1.83) |  | 224/496 | **1.53 (1.05 – 2.23)** | 294/496 | **1.39 (1.00 – 1.89)** |
| III | 84/163 | **2.07 (1.34 – 3.20)** | 114/163 | **1.94 (1.34 – 2.79)** |  | 51/74 | **2.24 (1.26 – 3.85)** | 68/74 | **2.06 (1.29 – 4.65)** |  | 135/237 | **2.19 (1.42 – 3.38)** | 182/237 | **2.01 (1.30 – 3.15)** |
| IV | 36/55 | **2.59 (1.57 – 4.29)** | 45/55 | **2.12 (1.36 – 3.31)** |  | 26/31 | **2.91 (1.44 – 5.73)** | 30/31 | **3.29 (1.51 – 7.10)** |  | 62/86 | **2.84 (1.53 – 4.69)** | 75/86 | **2.95 (1.44 – 5.89)** |
| Chemotherapy^c^ |  |  |  |  |  |  |  |  |  |  |  |  |  |  |
| No | 58/92 | Reference | 79/92 | Reference |  | 51/64 | Reference | 58/64 | Reference |  | 109/156 | Reference | 137/156 | Reference |
| Yes | 171/410 | **0.70 (0.52 – 0.96)** | 231/410 | **0.68 (0.44 – 0.93)** |  | 80/167 | **0.77 (0.61 – 0.98)** | 96/167 | **0.73 (0.47 – 0.98)** |  | 251/577 | **0.71 (0.53 – 0.87)** | 327/577 | **0.69 (0.46 – 0.86)** |
| Note: Bold values denote P ≤ 0.05.  ^a^ Adjusted by age, sex, tumor site, tumor size, differentiation, TNM stage, and chemotherapy where appropriate.  ^b^ Unknown differentiation were censored due to the small number of subjects in this subgroup.  ^c^ Only including stage II and stage III GC patients. | | | | | | | | | | | | | | |

| **Supplementary Table 3**. Association of polymorphisms in circadian negative-feedback loop genes with clinical outcomes in GC patients. | | | | | | | | | | | | | | |  | |
| --- | --- | --- | --- | --- | --- | --- | --- | --- | --- | --- | --- | --- | --- | --- | --- | --- |
| Gene | SNP | Genotype/Best fitting model | Training set | | | |  | Validation set | | |  | Pooled analysis | | |  | |
|  |  |  | Events^a^/Total | HR^b^ (95% CI) | *P* | Bootstrap^c^ (*P* < 0.05) |  | Events^a^/Total | HR^b^ (95% CI) | *P* |  | Events^a^/  Total | HR^b^ (95% CI) | *P* |  | |
| Overall Survival | |  |  |  |  |  |  |  |  |  |  |  |  |  |  |  |
| CRY1 | rs3809236 | CC | 176/429 | Reference |  |  |  | 106/198 | Reference |  |  | 282/627 | Reference |  |  | |
|  |  | CT | 103/237 | 1.03 (0.38 - 2.77) | 0.697 |  |  | 62/109 | 1.09 (0.44 - 1.63) | 0.761 |  | 165/346 | 0.94 (0.48 - 1.43) | 0.622 |  | |
|  |  | TT | 21/38 | 0.92 (0.33 - 2.55) | 0.297 |  |  | 14/19 | 0.95 (0.41 - 1.47) | 0.389 |  | 35/57 | 0.96 (0.39 - 1.56) | 0.168 |  | |
|  |  | Dominant |  | 0.85 (0.64 - 1.09) | 0.501 |  |  |  | 0.96 (0.75 - 1.21) | 0.713 |  |  | 0.92 (0.69 - 1.28) | 0.579 |  | |
|  | rs1056560 | TT | 188/379 | Reference |  |  |  | 120/176 | Reference |  |  | 308/555 | Reference |  |  | |
|  |  | TG | 99/274 | 0.76 (0.54 - 0.90) | 0.031 |  |  | 56/127 | 0.75 (0.44 - 0.92) | 0.028 |  | 155/401 | 0.69 (0.34 - 0.83) | 0.002 |  | |
|  |  | GG | 12/48 | 0.78 (0.55 - 0.92) | 0.038 |  |  | 6/22 | 0.79 (0.41 - 0.97) | 0.047 |  | 18/70 | 0.68 (0.31 - 0.87) | 0.004 |  | |
|  |  | Additive |  | **0.72 (0.58 - 0.88)** | **0.021** | 96 |  |  | **0.74 (0.46 - 0.90)** | **0.023** |  |  | **0.65 (0.34 - 0.87)** | **0.001** |  | |
| CRY2 | rs6798 | TT | 79/171 | Reference |  |  |  | 49/79 | Reference |  |  | 128/250 | Reference |  |  | |
|  |  | TC | 137/357 | 0.78 (0.56 - 1.10) | 0.272 |  |  | 84/165 | 0.85 (0.56 - 1.28) | 0.382 |  | 221/522 | 0.84 (0.51 - 1.12) | 0.159 |  | |
|  |  | CC | 77/169 | 0.94 (0.64 - 1.39) | 0.943 |  |  | 47/80 | 0.98 (0.74 - 1.39) | 0.834 |  | 124/249 | 0.97 (0.63 - 1.89) | 0.858 |  | |
|  |  | Dominant |  | 0.88 (0.67 - 1.16) | 0.422 |  |  |  | 0.97 (0.57 - 1.61) | 0.869 |  |  | 0.95 (0.64 - 1.42) | 0.287 |  | |
|  | rs2292910 | GG | 159/360 | Reference |  |  |  | 96/166 | Reference |  |  | 255/526 | Reference |  |  | |
|  |  | GA | 111/284 | 0.78 (0.57 - 1.07) | 0.699 |  |  | 67/131 | 0.87 (0.55 - 1.41) | 0.534 |  | 178/414 | 0.79 (0.43 - 1.35) | 0.308 |  | |
|  |  | AA | 30/59 | 0.97 (0.76 - 1.24) | 0.399 |  |  | 19/28 | 1.29 (0.71 - 1.79) | 0.621 |  | 49/87 | 1.27 (0.69 - 1.54) | 0.440 |  | |
|  |  | Dominant |  | 0.95 (0.76 - 1.19) | 0.652 |  |  |  | 1.12 (0.51 - 2.50) | 0.725 |  |  | 1.07 (0.72 - 1.58) | 0.695 |  | |
| PER1 | rs2735611 | CC | 134/312 | Reference |  |  |  | 81/142 | Reference |  |  | 215/454 | Reference |  |  | |
|  |  | CT | 127/314 | 1.03 (0.80 - 1.33) | 0.684 |  |  | 77/146 | 0.95 (0.64 - 1.53) | 0.692 |  | 204/460 | 0.91 (0.62 - 1.33) | 0.578 |  | |
|  |  | TT | 38/73 | 0.86 (0.54 - 1.39) | 0.393 |  |  | 23/36 | 0.88 (0.52 - 1.48) | 0.706 |  | 61/109 | 0.85 (0.48 - 1.37) | 0.353 |  | |
|  |  | Dominant |  | 0.87 (0.68 - 1.11) | 0.275 |  |  |  | 0.92 (0.58 - 1.48) | 0. 841 |  |  | 0.95 (0.65 - 1.57) | 0.881 |  | |
|  | rs3027178 | AA | 121/352 | Reference |  |  |  | 73/163 | Reference |  |  | 194/515 | Reference |  |  | |
|  |  | AC | 147/292 | 1.66 (1.21 - 2.15) | 0.009 |  |  | 89/135 | 1.55 (1.04 - 1.99) | 0.048 |  | 236/427 | 1.75 (1.24 - 2.92) | 0.001 |  | |
|  |  | CC | 29/56 | 1.19 (0.85 - 1.64) | 0.102 |  |  | 19/27 | 1.37 (0.95 - 1.97) | 0.170 |  | 48/83 | 1.49 (1.09 - 1.93) | 0.031 |  | |
|  |  | Dominant |  | **1.72 (1.19 - 2.35)** | **0.001** | 100 |  |  | **1.54 (1.07 - 1.98)** | **0.034** |  |  | **1.71 (1.25 - 2.34)** | **<0.001** |  | |
| PER2 | rs2304669 | AA | 225/549 | Reference |  |  |  | 137/253 | Reference |  |  | 362/802 | Reference |  |  | |
|  |  | AG | 66/142 | 0.90 (0.66 - 1.21) | 0.456 |  |  | 40/66 | 1.14 (0.72 - 1.52) | 0.619 |  | 106/208 | 1.18 (0.63 - 1.49) | 0.369 |  | |
|  |  | GG | 5/9 | 0.97 (0.36 - 2.62) | 0.588 |  |  | 3/5 | 1.08 (0.69 - 1.41) | 0.889 |  | 8/14 | 1.13 (0.61 - 1.56) | 0.598 |  | |
|  |  | Dominant |  | 0.92 (0.70 - 1.20) | 0.403 |  |  |  | 1.12 (0.83 - 1.58) | 0.612 |  |  | 1.16 (0.70 - 1.52) | 0.326 |  | |
|  | rs934945 | CC | 90/196 | Reference |  |  |  | 55/91 | Reference |  |  | 145/287 | Reference |  |  | |
|  |  | CT | 137/329 | 0.98 (0.72 - 1.33) | 0.548 |  |  | 83/153 | 0.92 (0.64 - 1.51) | 0.621 |  | 220/482 | 0.95 (0.71 - 1.48) | 0.436 |  | |
|  |  | TT | 71/175 | 0.71 (0.33 - 1.52) | 0.514 |  |  | 43/81 | 0.84 (0.56 - 1.45) | 0.610 |  | 114/256 | 0.79 (0.42 - 1.33) | 0.406 |  | |
|  |  | Dominant |  | 0.76 (0.56-1.25) | 0.482 |  |  |  | 0.91 (0.62 - 1.47) | 0.862 |  |  | 0.89 (0.58-1.35) | 0.933 |  | |
| PER3 | rs228729 | GG | 143/364 | Reference |  |  |  | 88/168 | Reference |  |  | 231/532 | Reference |  |  | |
|  |  | GA | 123/295 | 0.96 (0.74 - 1.25) | 0.683 |  |  | 78/136 | 1.21 (0.65 - 1.72) | 0.624 |  | 201/431 | 1.36 (0.82 - 1.53) | 0.331 |  | |
|  |  | AA | 34/41 | 1.90 (1.26 - 2.85) | 0.004 |  |  | 15/20 | 1.38 (0.79 - 1.94) | 0.216 |  | 49/61 | 1.75 (1.21 - 3.35) | 0.008 |  | |
|  |  | Recessive |  | **1.93 (1.31 - 2.85)** | **0.003** | 98 |  |  | 1.39 (0.82 - 1.97) | 0.170 |  |  | **1.79 (1.29 - 2.93)** | **0.003** |  | |
|  | rs228669 | GG | 154/383 | Reference |  |  |  | 93/177 | Reference |  |  | 247/560 | Reference |  |  | |
|  |  | GA | 123/275 | 1.17 (0.89 - 1.48) | 0.461 |  |  | 75/127 | 1.22 (0.82 - 1.61) | 0.547 |  | 198/402 | 1.07 (0.66 - 1.39) | 0.340 |  | |
|  |  | AA | 21/43 | 1.33 (0.82 - 2.15) | 0.491 |  |  | 13/21 | 1.18 (0.71 - 1.38) | 0.662 |  | 34/64 | 1.12 (0.61 - 1.45) | 0.409 |  | |
|  |  | Dominant |  | 1.15 (0.95 - 1.40) | 0.391 |  |  |  | 1.14 (0.79 - 1.42) | 0.506 |  |  | 1.19 (0.86 - 1.51) | 0.275 |  | |
|  | rs2640908 | TT | 71/182 | Reference |  |  |  | 43/84 | Reference |  |  | 114/266 | Reference |  |  | |
|  |  | TC | 135/353 | 1.06 (0.78 - 1.42) | 0.722 |  |  | 82/163 | 0.98 (0.73 - 1.33) | 0.940 |  | 217/516 | 0.90 (0.68 - 1.64) | 0.891 |  | |
|  |  | CC | 94/169 | 1.39 (0.98 - 1.95) | 0.062 |  |  | 57/79 | 1.36 (0.92 - 1.84) | 0.179 |  | 151/248 | 1.39 (0.96 - 1.91) | 0.088 |  | |
|  |  | Recessive |  | 1.44 (0.98 - 1.96) | 0.076 |  |  |  | 1.31 (0.93 - 1.86) | 0.127 |  |  | 1.27 (0.95 - 1.62) | 0.113 |  | |
|  | rs172933 | CC | 169/411 | Reference |  |  |  | 101/190 | Reference |  |  | 270/601 | Reference |  |  | |
|  |  | CT | 106/248 | 1.03 (0.76 - 1.39) | 0.793 |  |  | 64/115 | 1.16 (0.81 - 1.72) | 0.817 |  | 170/363 | 1.07 (0.69 - 1.44) | 0.725 |  | |
|  |  | TT | 24/41 | 1.15 (0.68 - 1.96) | 0.194 |  |  | 16/21 | 1.28 (0.78 - 1.62) | 0.307 |  | 40/62 | 1.31 (0.97 - 1.75) | 0.092 |  | |
|  |  | Recessive |  | 1.21 (0.74 - 1.44) | 0.203 |  |  |  | 1.17 (0.64 - 1.51) | 0.320 |  |  | 1.25 (0.98 - 1.57) | 0.062 |  | |
|  | rs2859390 | AA | 197/459 | Reference |  |  |  | 119/213 | Reference |  |  | 316/672 | Reference |  |  | |
|  |  | AG | 92/216 | 0.98 (0.72 - 1.33) | 0.960 |  |  | 56/100 | 1.03 (0.80 - 1.38) | 0.991 |  | 148/316 | 0.95 (0.78 - 1.42) | 0.973 |  | |
|  |  | GG | 10/26 | 0.71 (0.33 - 1.52) | 0.774 |  |  | 6/12 | 0.96 (0.78 - 1.29) | 0.829 |  | 16/38 | 0.93 (0.71 - 1.34) | 0.718 |  | |
|  |  | Dominant |  | 0.92 (0.72 - 1.18) | 0.516 |  |  |  | 0.98 (0.73 - 1.31) | 0.962 |  |  | 0.94 (0.69 - 1.43) | 0.898 |  | |
| Relapse-free Survival | |  |  |  |  |  |  |  |  |  |  |  |  |  |  |  |
| CRY1 | rs3809236 | CC | 241/429 | Reference |  |  |  | 124/198 | Reference |  |  | 365/627 | Reference |  |  | |
|  |  | CT | 157/237 | 1.16 (0.74 - 1.55) | 0.207 |  |  | 79/109 | 1.13 (0.61 - 1.58) | 0.434 |  | 236/346 | 1.12 (0.70 - 1.49) | 0.139 |  | |
|  |  | TT | 25/38 | 1.19 (0.53 - 1.64) | 0.558 |  |  | 15/19 | 1.21 (0.66 - 1.71) | 0.524 |  | 40/57 | 1.17 (0.64 - 1.53) | 0.388 |  | |
|  |  | Dominant |  | 1.25 (0.78 - 1.57) | 0.189 |  |  |  | 1.29 (0.82 - 1.81) | 0.370 |  |  | 1.12 (0.70 - 1.45) | 0.551 |  | |
|  | rs1056560 | TT | 230/379 | Reference |  |  |  | 129/176 | Reference |  |  | 359/555 | Reference |  |  | |
|  |  | TG | 167/274 | 0.96 (0.80 - 1.21) | 0.633 |  |  | 77/127 | 0.86 (0.60 - 1.24) | 0.305 |  | 244/401 | 0.93 (0.75 - 1.22) | 0.563 |  | |
|  |  | GG | 25/48 | 0.83 (0.59 - 1.41) | 0.557 |  |  | 11/22 | 0.83 (0.58 - 1.21) | 0.321 |  | 36/70 | 0.82 (0.62 - 1.19) | 0.288 |  | |
|  |  | Additive |  | 0.84 (0.66 - 1.34) | 0.356 |  |  |  | 0.88 (0.66 - 1.18) | 0.399 |  |  | 0.88 (0.64 - 1.15) | 0.149 |  | |
| CRY2 | rs6798 | TT | 111/171 | Reference |  |  |  | 57/79 | Reference |  |  | 168/250 | Reference |  |  | |
|  |  | TC | 200/357 | 1.06 (0.78 - 1.43) | 0.328 |  |  | 103/165 | 0.91 (0.65 - 1.38) | 0.499 |  | 303/522 | 1.08 (0.66 - 1.52) | 0.234 |  | |
|  |  | CC | 109/169 | 1.01 (0.78 - 1.32) | 0.920 |  |  | 56/80 | 1.04 (0.76 - 1.23) | 0.902 |  | 165/249 | 0.99 (0.67 - 1.35) | 0.921 |  | |
|  |  | Dominant |  | 0.96 (0.75 - 1.22) | 0.480 |  |  |  | 0.93 (0.62 - 1.46) | 0.599 |  |  | 0.96 (0.68 - 1.27) | 0.389 |  | |
|  | rs2292910 | GG | 219/360 | Reference |  |  |  | 112/166 | Reference |  |  | 331/526 | Reference |  |  | |
|  |  | GA | 167/284 | 0.95 (0.76 - 1.18) | 0.794 |  |  | 83/131 | 0.89 (0.55 - 1.36) | 0.735 |  | 250/414 | 0.90 (0.52 - 1.34) | 0.699 |  | |
|  |  | AA | 37/59 | 1.03 (0.71 - 1.51) | 0.893 |  |  | 23/28 | 1.14 (0.69 - 1.57) | 0.521 |  | 60/87 | 1.08 (0.67 - 1.45) | 0.614 |  | |
|  |  | Dominant |  | 0.98 (0.84 - 1.16) | 0.855 |  |  |  | 0.92 (0.64 - 1.33) | 0.945 |  |  | 0.91 (0.67 - 1.22) | 0.705 |  | |
| PER1 | rs2735611 | CC | 182/312 | Reference |  |  |  | 93/142 | Reference |  |  | 275/454 | Reference |  |  | |
|  |  | CT | 190/314 | 1.01 (0.71 - 1.44) | 0.780 |  |  | 97/146 | 1.03 (0.77 - 1.38) | 0.939 |  | 287/460 | 1.07 (0.63 - 1.51) | 0.706 |  | |
|  |  | TT | 49/73 | 0.99 (0.69 - 1.41) | 0.497 |  |  | 26/36 | 1.09 (0.66 - 1.49) | 0.736 |  | 75/109 | 1.09 (0.65 - 1.48) | 0.411 |  | |
|  |  | Dominant |  | 0.99 (0.84 - 1.16) | 0.647 |  |  |  | 1.05 (0.67 - 1.40) | 0.859 |  |  | 1.08 (0.54 - 1.38) | 0.556 |  | |
|  | rs3027178 | AA | 181/352 | Reference |  |  |  | 99/163 | Reference |  |  | 280/515 | Reference |  |  | |
|  |  | AC | 199/292 | 1.39 (1.11 - 1.75) | 0.030 |  |  | 97/135 | 1.35 (0.91 - 1.87) | 0.115 |  | 296/427 | 1.35 (0.97 - 1.75) | 0.086 |  | |
|  |  | CC | 39/56 | 1.12 (0.75 - 1.67) | 0.182 |  |  | 21/27 | 1.27 (0.84 - 1.73) | 0.330 |  | 60/83 | 1.24 (0.89 - 1.66) | 0.123 |  | |
|  |  | Dominant |  | 1.24 (0.96 - 1.73) | 0.092 |  |  |  | 1.29 (0.98 - 1.46) | 0.095 |  |  | 1.27 (0.98 - 1.39) | 0.064 |  | |
| PER2 | rs2304669 | AA | 318/549 | Reference |  |  |  | 165/253 | Reference |  |  | 483/802 | Reference |  |  | |
|  |  | AG | 94/142 | 0.96 (0.74 - 1.25) | 0.375 |  |  | 49/66 | 1.05 (0.68 - 1.39) | 0.544 |  | 143/208 | 0.97 (0.70 - 1.33) | 0.281 |  | |
|  |  | GG | 7/9 | 1.24 (0.55 - 2.78) | 0.561 |  |  | 4/5 | 1.13 (0.72 - 1.71) | 0.763 |  | 11/14 | 1.26 (0.63 - 2.35) | 0.512 |  | |
|  |  | Dominant |  | 0.99 (0.80 - 1.26) | 0.326 |  |  |  | 1.08 (0.62 - 1.83) | 0.515 |  |  | 1.07 (0.68 - 1.52) | 0.610 |  | |
|  | rs934945 | CC | 136/196 | Reference |  |  |  | 70/91 | Reference |  |  | 206/287 | Reference |  |  | |
|  |  | CT | 197/329 | 1.19 (0.83 - 1.44) | 0.304 |  |  | 102/153 | 0.91 (0.53 - 1.52) | 0.483 |  | 299/482 | 0.97 (0.61 - 1.43) | 0.213 |  | |
|  |  | TT | 86/175 | 0.86 (0.69 - 1.05) | 0.098 |  |  | 45/81 | 0.85 (0.56 - 1.24) | 0.183 |  | 131/256 | 0.83 (0.54 - 1.04) | 0.096 |  | |
|  |  | Dominant |  | 0.93 (0.61 - 1.19) | 0.114 |  |  |  | 0.94 (0.68 - 1.27) | 0.780 |  |  | 0.89 (0.56 - 1.46) | 0.085 |  | |
| PER3 | rs228729 | GG | 201/364 | Reference |  |  |  | 107/168 | Reference |  |  | 308/532 | Reference |  |  | |
|  |  | GA | 182/295 | 0.98 (0.78 - 1.22) | 0.501 |  |  | 92/136 | 0.95 (0.69 - 1.37) | 0.742 |  | 274/431 | 0.96 (0.70 - 1.39) | 0.374 |  | |
|  |  | AA | 36/41 | 1.37 (0.99 - 2.33) | 0.054 |  |  | 17/20 | 1.26 (0.83 - 1.74) | 0.412 |  | 53/61 | 1.35 (0.97 - 1.94) | 0.072 |  | |
|  |  | Recessive |  | 1.21 (0.97 - 1.72) | 0.072 |  |  |  | 1.17 (0.72 - 1.44) | 0.598 |  |  | 1.34 (0.93 - 2.10) | 0.172 |  | |
|  | rs228669 | GG | 216/383 | Reference |  |  |  | 111/177 | Reference |  |  | 327/560 | Reference |  |  | |
|  |  | GA | 174/275 | 1.15 (0.89 - 1.48) | 0.372 |  |  | 90/127 | 1.07 (0.68 - 1.34) | 0.505 |  | 264/402 | 1.09 (0.78 - 1.25) | 0.265 |  | |
|  |  | AA | 30/43 | 1.43 (0.95 - 2.15) | 0.399 |  |  | 16/21 | 1.21 (0.73 - 1.91) | 0.581 |  | 46/64 | 1.18 (0.69 - 1.41) | 0.311 |  | |
|  |  | Dominant |  | 1.28 (0.98 - 1.59) | 0.297 |  |  |  | 1.16 (0.70 - 1.65) | 0.449 |  |  | 1.21 (0.76 - 1.42) | 0.197 |  | |
|  | rs2640908 | TT | 110/182 | Reference |  |  |  | 56/84 | Reference |  |  | 166/266 | Reference |  |  | |
|  |  | TC | 208/353 | 1.13 (0.87 - 1.46) | 0.865 |  |  | 102/163 | 1.16 (0.82 - 1.45) | 0.767 |  | 310/516 | 1.19 (0.84 - 1.42) | 0.756 |  | |
|  |  | CC | 105/169 | 1.28 (0.95 - 1.72) | 0.874 |  |  | 60/79 | 1.34 (0.75 - 1.81) | 0.592 |  | 165/248 | 1.38 (0.85 - 1.85) | 0.650 |  | |
|  |  | Recessive |  | 1.18 (0.93 - 1.51) | 0.757 |  |  |  | 1.25 (0.82 - 1.78) | 0.389 |  |  | 1.24 (0.83 - 1.75) | 0.443 |  | |
|  | rs172933 | CC | 231/411 | Reference |  |  |  | 119/190 | Reference |  |  | 350/601 | Reference |  |  | |
|  |  | CT | 160/248 | 1.12 (0.86 - 1.45) | 0.291 |  |  | 82/115 | 1.08 (0.78 - 1.34) | 0.485 |  | 242/363 | 1.14 (0.83 - 1.55) | 0.205 |  | |
|  |  | TT | 28/41 | 1.13 (0.70 - 1.82) | 0.451 |  |  | 18/21 | 1.36 (0.85 - 1.87) | 0.357 |  | 46/62 | 1.27 (0.85 - 1.49) | 0.239 |  | |
|  |  | Recessive |  | 1.26 (0.76 - 1.77) | 0.579 |  |  |  | 1.19 (0.77 - 1.62) | 0.430 |  |  | 1.21 (0.86 - 1.70) | 0.347 |  | |
|  | rs2859390 | AA | 274/459 | Reference |  |  |  | 142/213 | Reference |  |  | 416/672 | Reference |  |  | |
|  |  | AG | 129/216 | 0.95 (0.73 - 1.24) | 0.997 |  |  | 67/100 | 0.97 (0.68 - 1.32) | 0.979 |  | 196/316 | 0.91 (0.67 - 1.33) | 0.986 |  | |
|  |  | GG | 15/26 | 0.59 (0.29 - 1.19) | 0.918 |  |  | 8/12 | 0.99 (0.79 - 1.28) | 0.982 |  | 23/38 | 0.94 (0.72 - 1.43) | 0.934 |  | |
|  |  | Dominant |  | 0.88 (0.71 - 1.09) | 0.980 |  |  |  | 0.97 (0.58 - 1.64) | 0.981 |  |  | 0.93 (0.68 - 1.37) | 0.995 |  | |
| Note: The significant values were shown in boldface (*P* < 0.05).  HR indicates hazard ratio; CI, confidence interval; TFBS, transcription factor binding site.  ^a^ Numbers may not add up to 100% of available subjects because of missing genotyping data.  ^b^ Adjusted by age, sex, tumor site, tumor size, differentiation, TNM stage, and chemotherapy where appropriate.  ^c^ Bootstrap analysis was performed using 100 replicates to determine statistics support. | | | | | | | | | | | | | | |  | |

**Supplementary Table 4**. Association of significant SNPs with overall survival of GC patients stratified by chemotherapy.

| SNP | Genotype | In patients with chemotherapy^a^ | | |  | In patients without chemotherapy^a^ | | |
| --- | --- | --- | --- | --- | --- | --- | --- | --- |
|  |  | Deaths/Total^b^ | HR^c^ (95%CI) | *P* |  | Deaths/Total^b^ | HR^c^ (95%CI) | *P* |
| Training set |  |  |  |  |  |  |  |  |
| rs1056560 | TT | 116/221 | Reference |  |  | 36/50 | Reference |  |
|  | TG+GG | 55/189 | 0.55 (0.40 – 0.77) | 0.001 |  | 21/41 | 0.78 (0.52 – 1.19) | 0.143 |
| rs3027178 | AA | 69/206 | Reference |  |  | 21/47 | Reference |  |
|  | AC+CC | 101/203 | 1.51 (1.12 – 2.52) | 0.009 |  | 35/43 | 1.43 (1.05 – 2.46) | 0.038 |
| rs228729 | GG+GA | 156/385 | Reference |  |  | 53/86 | Reference |  |
|  | AA | 15/25 | 1.44 (1.05 – 1.96) | 0.037 |  | 5/6 | 1.41 (1.01 – 1.96) | 0.046 |
| Validation set |  |  |  |  |  |  |  |  |
| rs1056560 | TT | 53/88 | Reference |  |  | 32/35 | Reference |  |
|  | TG+GG | 27/79 | 0.60 (0.34 – 0.84) | 0.005 |  | 19/29 | 0.81 (0.56 – 1.25) | 0.384 |
| rs3027178 | AA | 31/86 | Reference |  |  | 22/33 | Reference |  |
|  | AC+CC | 49/81 | 1.38 (1.05 – 1.87) | 0.021 |  | 29/31 | 1.27 (1.02 – 1.46) | 0.041 |
| rs228729 | GG+GA | 72/156 | Reference |  |  | 47/60 | Reference |  |
|  | AA | 7/10 | 1.38 (1.03 – 1.76) | 0.041 |  | 4/4 | 1.35 (0.99 – 1.93) | 0.057 |
| Pooled analysis |  |  |  |  |  |  |  |  |
| rs1056560 | TT | 169/309 | Reference |  |  | 68/85 | Reference |  |
|  | TG+GG | 82/268 | 0.58 (0.39 – 0.83) | 0.005 |  | 40/70 | 0.80 (0.59 – 1.09) | 0.189 |
| rs3027178 | AA | 100/292 | Reference |  |  | 43/80 | Reference |  |
|  | AC+CC | 150/284 | 1.55 (1.12 – 2.07) | 0.004 |  | 64/74 | 1.46 (1.04 – 1.91) | 0.046 |
| rs228729 | GG+GA | 228/541 | Reference |  |  | 100/146 | Reference |  |
|  | AA | 22/35 | 1.35 (1.06 – 1.87) | 0.028 |  | 9/10 | 1.44 (1.02 – 1.95) | 0.046 |

Note: The significant values were shown in boldface (*P* < 0.05). HR, hazard ratio; CI, confidence interval.

^a^ Only including stage II and stage III GC patients.

^b^ Numbers may not add up to 100% of available subjects because of missing genotyping data.

^c^ Adjusted by age, sex, tumor site, tumor size, differentiation, TNM stage, and chemotherapy where appropriate.
